# Utility of the Base Editing System for Introducing Drug-Resistant Gene Mutations Into Human Leukemia Cellular Models

**DOI:** 10.7759/cureus.81889

**Published:** 2025-04-08

**Authors:** Thao Nguyen, Minori Tamai, Shinichi Fujisawa, Akiko Nagamachi, Keiko Kagami, Chiaki Komatsu, Shotaro Iwamoto, Toshiya Inaba, Takanori Teshima, Takeshi Inukai

**Affiliations:** 1 Pediatrics, University of Yamanashi, Chuo, JPN; 2 Pediatrics, Global Leukemia Cell-Line Assembly Network, University of Yamanashi, Chuo, JPN; 3 Laboratory and Transfusion Medicine, Hokkaido University Hospital, Sapporo, JPN; 4 Molecular Oncology and Leukemia Program Project, Research Institute for Radiation Biology and Medicine, Hiroshima University, Hiroshima, JPN; 5 Global Leukemia Cell-Line Assembly Network, University of Yamanashi, Chuo, JPN; 6 Pediatrics, Mie University Graduate School of Medicine, Tsu, JPN

**Keywords:** base editing, bcr::abl1, drug resistance, genome editing, leukemia, mutation, tp53

## Abstract

Background

Recent genomic analyses of poor prognostic and relapsed leukemia have revealed the involvement of diverse gene mutations in treatment resistance. These gene mutations are classified into two groups: mutations involving resistance to specific agents such as the *BCR::ABL1* fusion gene mutations (typically T315I mutation) in tyrosine kinase inhibitor (TKI) resistance and those involving the resistance to diverse therapeutic modalities such as the *TP53* gene mutations. In the latter type, although their associations with drug resistance have been clinically demonstrated, the direct association with resistance to each therapeutic modality remains to be fully elucidated. To overcome treatment resistance induced by these gene mutations, appropriate leukemic cellular models are urgently required. Using the cytidine base editing (CBE) system, we introduced two types of mutations through C-to-T transition into human leukemia cell lines and evaluated their significance in the drug sensitivities.

Methods

We applied the CBE system to introduce the T315I (ACT to ATT) mutation of the *BCR::ABL1* fusion gene in a human Philadelphia chromosome-positive leukemia cell line and to introduce the T125M (ACG to ATG) mutation of the *TP53* gene in a human B-cell precursor acute lymphoblastic leukemia (BCP-ALL) cell line.

Results

We first confirmed an introduction of the T315I mutation in one of four *BCR::ABL1* alleles as a result of base editing in the obtained TKI-resistant subline. We also identified the additional C-to-T transition at adjacent codon 314 (ATC), which resulted in a silent mutation, in the same allele. We next confirmed that the obtained subline acquired the T125M mutation of the *TP53* gene without additional C-to-T transition. In the T125M subline, transcriptional activities of the p53 protein were disrupted and the sensitivities to diverse chemotherapeutic drugs and irradiation were reduced.

Conclusion

Our observations demonstrated the utility of the CBE system for introducing specific nucleotide transitions into human leukemia cell lines.

## Introduction

Therapeutic outcomes in leukemia patients have dramatically improved since the introduction of combined chemotherapy and the development of specific inhibitors. Recent genomic analyses of poor prognostic and relapsed leukemia have revealed the involvement of diverse gene mutations in drug resistance [1,2]. These gene mutations are largely classified into two types: a mutation involving resistance to specific agents and a mutation involving resistance to multiple agents with different pharmacological properties. The former type includes the *BCR::ABL1* fusion gene mutation (typically T315I mutation) in specific resistance to tyrosine kinase inhibitors (TKIs), the glucocorticoid receptor (*NR3C1*) gene mutation in specific resistance to glucocorticoids [3], and the *NT5C2* [4,5] and* PRPS1* [5,6] gene mutations in specific resistance to mercaptopurines. In these mutations, their functional associations with drug resistance are relatively straightforward. The latter type includes *TP53* gene mutation [1] and Ras pathway gene mutations [7]. In these mutations, although their associations with drug-resistant properties have been clinically demonstrated, the direct association with resistance to each therapeutic modality remains to be fully elucidated. In this context, to overcome the drug resistance induced by the latter type of mutation, the establishment of proper leukemic cellular models is urgently required. 

To introduce a specific mutation into the intrinsic wild-type gene, homologous, directed repair (HDR)-induced by the clustered regularly interspaced short palindromic repeats (CRISPR)/Cas9 system, is potentially applicable [8-10]. However, despite its utility, DNA double-strand break (DSB) induced by the CRISPR/Cas9 system dominantly activates the non-homologous end joining (NHEJ) pathway, which results in unexpected insertion/deletion (indel), rather than the HDR pathway. Moreover, the intrinsic DNA-repair system for introducing HDR is largely disrupted in many leukemia cell lines [10].

Under these circumstances, to overcome the above limitations of the traditional CRISPR/Cas9 system, the newly developed base editing system is an attractive genome editing tool [11,12]. The base editing system enables the introduction of specific single-nucleotide variants without generating DSB by using a cytidine deaminase-fused nickase Cas9 (nCas9). In the cytidine base editing (CBE) system, the cytidine deaminase converts cytidine (C) to thymidine (T) on the nucleotides located in the “editing window” [11,12]. When the target C nucleotide is located in the editing window and if any other C nucleotides in the editing window are supposed to induce silent mutations, the CBE system could be useful to induce C-to-T (or guanine (G)-to-adenine (A)) transition. Although *TP53* and Ras pathway mutations are clinically linked to multi-drug resistance [1,7], their mechanistic roles remain poorly understood due to a lack of isogenic cellular models. Thus, the base editing system could offer a precise solution to address this gap, when it is applicable for introducing such mutations.

In the present study, to verify the utility of the CBE system, we first tried to introduce the T315I (ACT to ATT) mutation of the *BCR::ABL1* fusion gene, which is the most important *BCR::ABL1* mutation, in a human Ph+ myeloid leukemia cell line. We successfully obtained the T315I subline, which expectedly showed TKI resistance, indicating the utility of the CBE system for introducing specific C-to-T transition in human leukemia cell lines. Subsequently, to investigate the drug-resistant phenotype induced by the *TP53* mutation, we tried to introduce the T125M (ACG to ATG) mutation of the *TP53* gene, which is one of the few C-to-T transition-type *TP53* mutations without neighboring C nucleotides that affect coding amino acid in the editing window, into a human B-cell precursor acute lymphoblastic leukemia (BCP-ALL) cell line with an intact *TP53* gene. We successfully obtained the T125M subline with the designed mutations and evaluated its drug-resistant phenotype.

## Materials and methods

Cell line and reagents

The TCCS cell line, which was established from a patient with myeloid blast crisis of a CML and has the p210 BCR::ABL1 protein [13], was provided by Prof. N. Komatsu (Juntendo University). The 697 cell line was established from a patient with BCP-ALL [14] and has the *TCF3::PBX1* fusion gene [15] and intact *TP53* gene [16]. The MB-IT cell line was established from the patient with BCP-ALL [17] and has the *ETV6::RUNX1 *fusion [3]. These cell lines were maintained in RPMI1640 medium supplemented with 10% fetal calf serum (FCS) in a humidified atmosphere of 5% CO_2 _at 37°C.

Dual-color fluorescence in situ hybridization analysis 

After 2 h of treatment with 0.1 µg/mL of KaryoMAX COLCEMID Solution (Thermo Fisher Scientific), the cells were exposed to 0.075 mol/L of KCl at 37°C for 15 min and fixed on slide glasses using a 3:1 methanol/glacial acetic acid solution three times. Subsequently, the air-dried slide samples were denatured at 75°C for 1 min and hybridized with LSI *BCR::ABL1* Dual Fusion, LSI ASS-*ABL* SpectrumOrange, and LSI *BCR* SpectrumGreen probes (Vysis/Abbott, Chicago, IL) at 37°C overnight. Analyses were performed using a CytoVision system (Applied Imaging, Santa Clara, CA).

Plasmid construction and transfection 

pCMV_BE4max_P2A_GFP (Addgene #112099) and MLM3636 (Addgene #43860) were provided by David Liu and Keith Joung, respectively. Numbers of the potential off-target sites were evaluated by Cas-OFFinder (http://www.rgenome.net/cas-offinder/) [18]. SgRNA oligomers for the introduction of *BCR::ABL1*-T315I mutation or *TP53*-T125M mutation (Table 1) were synthesized by Integrated DNA Technologies (Coralville, IA), then annealed and cloned into MLM3636 vector (Thermo Fisher Scientific). The Neon system (Thermo Fisher Scientific) was applied for transfection by electroporating 250 ng of MLM3636 plasmid expressing sgRNA and 750 ng of BE4max into 3 × 10^6^ cells. For the introduction of *BCR::ABL1*-T315I mutation, cells were pulsed at 1400 V for 30 msec, and the electroporated cells were then expanded for seven days without imatinib in RPMI 1640 medium supplemented with 10% FCS in a humidified atmosphere of 5% CO_2_ at 37°C, and subsequently cultured in the presence of imatinib (SelleckChem, Pittsburgh, PA) at 1 μM. For the introduction of the *TP53*-T125M mutation, cells were pulsed at 1500 V for 20 msec, and the electroporated cells were then expanded for seven days without nutlin-3a in RPMI 1640 medium supplemented with 10% FCS in a humidified atmosphere of 5% CO_2_ at 37°C, and subsequently incubated with 10 µM of nutlin-3a (SelleckChem).

**Table 1:** List of oligomers.

sgRNA-expression plasmid construction	Sequence (5'-3')
CBE-*BCR::ABL1*-T315I-sgRNA-F	acacgATCACTGAGTTCATGACCTA
CBE-*BCR::ABL1*-T315I-sgRNA-R	aaaaTAGGTCATGAACTCAGTGAT
CBE-*TP53*-T125M-F	acacgTGCACGGTCAGTTGCCCTGA
CBE-*TP53*-T125M-R	aaaaTCAGGGCAACTGACCGTGCA
Genomic PCR	
*ABL*-315-F	CCACACGAGCACAGTCTCAG
*ABL*-315-R	AACTCAGTGATGATATAGAACGG
*TP53*-125-F	TTGGGCAGTGCTCGCTTAGTGC
*TP53*-125-R	TCCTGCAACCCACTAGCGAGCT
Luciferase reporter plasmids construction	
p53-RE-F	gtacctgagctcTACAGAACATGTCTAAGCATGCTGTGCCTTGCCTGGACTTGCCTGGCCTTGCCTTgggc
p53-RE-R	tcgagcccAAGGCAAGGCCAGGCAAGTCCAGGCAAGGCACAGCATGCTTAGACATGTTCTGTAgagctcag
p53-dRE-F	gtacctgagctcTACAGAATATtTCTAAGTATtCTGTGCCTTGCCTGGATTTtCCTGGCTTTtCCTTgggc
p53-dRE-R	tcgagcccAAGGaAAAGCCAGGaAAATCCAGGCAAGGCACAGaATACTTAGAaATATTCTGTAgagctcag

PCR, TA cloning, and sequencing

Genomic DNA was extracted using the PureLink Genomic DNA Mini Kit (Thermo Fisher Scientific) and PCR amplification of genomic DNA was performed using the primers listed in Table 1. TA cloning was performed using a TOPO^TM^ TA Cloning^TM^ Kit (Invitrogen). Direct sequencing of each PCR product was performed using forward primers (Table 1) with the standard condition. Editing efficiency was evaluated by EditR software, an algorithm for predicting potential editing in a guide RNA region from single Sanger sequencing [19]. 

Drug sensitivity assay

In brief, 2.5 × 10^4^ cells were incubated for 72 h in RPMI 1640 medium supplemented with 10% FCS in a humidified atmosphere of 5% CO_2_ at 37°C with or without six different concentrations of each reagent as follows: imatinib (0.1 - 10000 nM, S2417, Selleck Chemicals, Tokyo, Japan), nilotinib (0.05 - 5000 nM, kindly provided by Dr. Tetuzo Tauchi, Tokyo Medical University), dasatinib (0.01 - 1000 nM, kindly provided by Dr. Tetuzo Tauchi, Tokyo Medical University), ponatinib (0.01 - 1000 nM, CS-0204, Chem Scene, Monmouth Junction, NJ), daunorubicin (0.1 - 1000 nM, 23541-50-6, Sigma-Aldrich, St. Louis, MO), cytarabine (1 - 1000 nM, C2035, Tokyo Chemical Industry, Tokyo, Japan), asparaginase (0.1 - 1000 IU/mL, provided by Kyowa Hakko Kirin, Tokyo, Japan), dexamethasone (0.2 - 200 nM, 50-02-2, Sigma-Aldrich), nutlin-3a (1 - 1000 nM, S8059, Selleck Chemicals), prednisolone (0.1 - 1000 nM, 53-03-2, Sigma-Aldrich), and vincristine (0.1 - 1000 nM, 2068-78-2, Sigma-Aldrich). Then, the cells were cultured for 5 h in the presence of alamarBlue (Bio-Rad Laboratories, Hercules, CA), and subsequently the absorbance at 570 nm was measured by a microplate spectrophotometer (Bio-Rad Laboratories) using 600 nm as a reference wavelength. The cellular viability was calculated by expressing the rates of optical densities of the wells with the agents in comparison with those of the wells without the agents. The IC50 values represent the median drug concentrations in triplicate experiments required to decrease the cellular viability of treated wells to 50% of that in untreated wells. In the case of TKI sensitivities, the median IC_50_ values were determined by three independent assays. The fitting procedure of the dose-response curve was performed by a polynomial fit using Excel software.

Luciferase assay 

The pGL3-RE reporter plasmid with four tandemly repeated p53 responsive elements (Table 1) and the pGL3-dRE reporter plasmid with the mutations in all four p53 responsive elements (Table 1) were created based on the pGL3-basic (Promega, E1751). The parental cells and T125M-subline were transfected with either of the pGL3-basic, pGL3 control (Promega, E1741), pGL3-RE, or pGL3-dRE vectors by applying lipofectamine 3000 (ThermoFisher Scientific L3000001). pTK-RL (Promega, E2231), a reporter plasmid of *Renilla* luciferase, was co-transfected for each transfection as internal control. One day after transfection, the cells were incubated with or without 10 μM of nutlin-3a for 6 h and harvested for the assay following the instructions by the manufacturer. The assay was performed in triplicate by applying the Dual-Luciferase Reporter Assay System (Promega, E1910).

Real-time reverse transcription (RT)-PCR analysis

Extraction of mRNA was performed by applying PureLink RNA Mini Kit (ThermoFisher Scientific), and synthesis of cDNA was performed by applying SuperPrep^TM^ Cell Lysis & RT Kit for qPCR (Toyobo, Tokyo, Japan, SCQ-101). Real-time RT-PCR analyses of *CDKN1A* (Hs00355782_m1, ThermoFisher Scientific) and *PUMA* (Hs00248075_m1, ThermoFisher Scientific) were performed by applying the TaqMan Probe Kit using *ACTB* (Hs99999903_m1, ThermoFisher Scientific) as an internal control following the manufacturer’s instructions.

Sensitivity to irradiation

Gamma-irradiation was performed by the Gammacell 40 Exactor (MDS Nordion, Ottawa, Canada) (0.77 Gy/min for 13.2 min). Then, a trypan blue dye exclusion assay was performed for the evaluation of living cell number and cellular viability.

Statistics

Jamovi software (version 2.3.16, Jamovi, Sydney, Australia) was applied for student t-tests.

## Results

Introduction of T315I mutation of *BCR::ABL1* fusion gene into myeloid cell line TCCS

**Figure 1:**
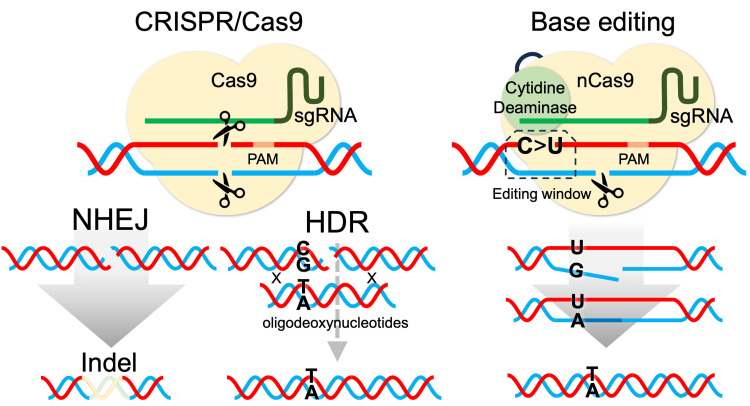
HDR mediated by CRISPR/Cas9 and CBE. Schematic representation of NHEJ (left pane) and HDR (middle panel) induced by the CRISPR/Cas9 system and CBE (right panel). NHEJ, non-homologous end joining; CBE, cytidine base editing; HDR, homologous-directed repair

**Figure 2:**
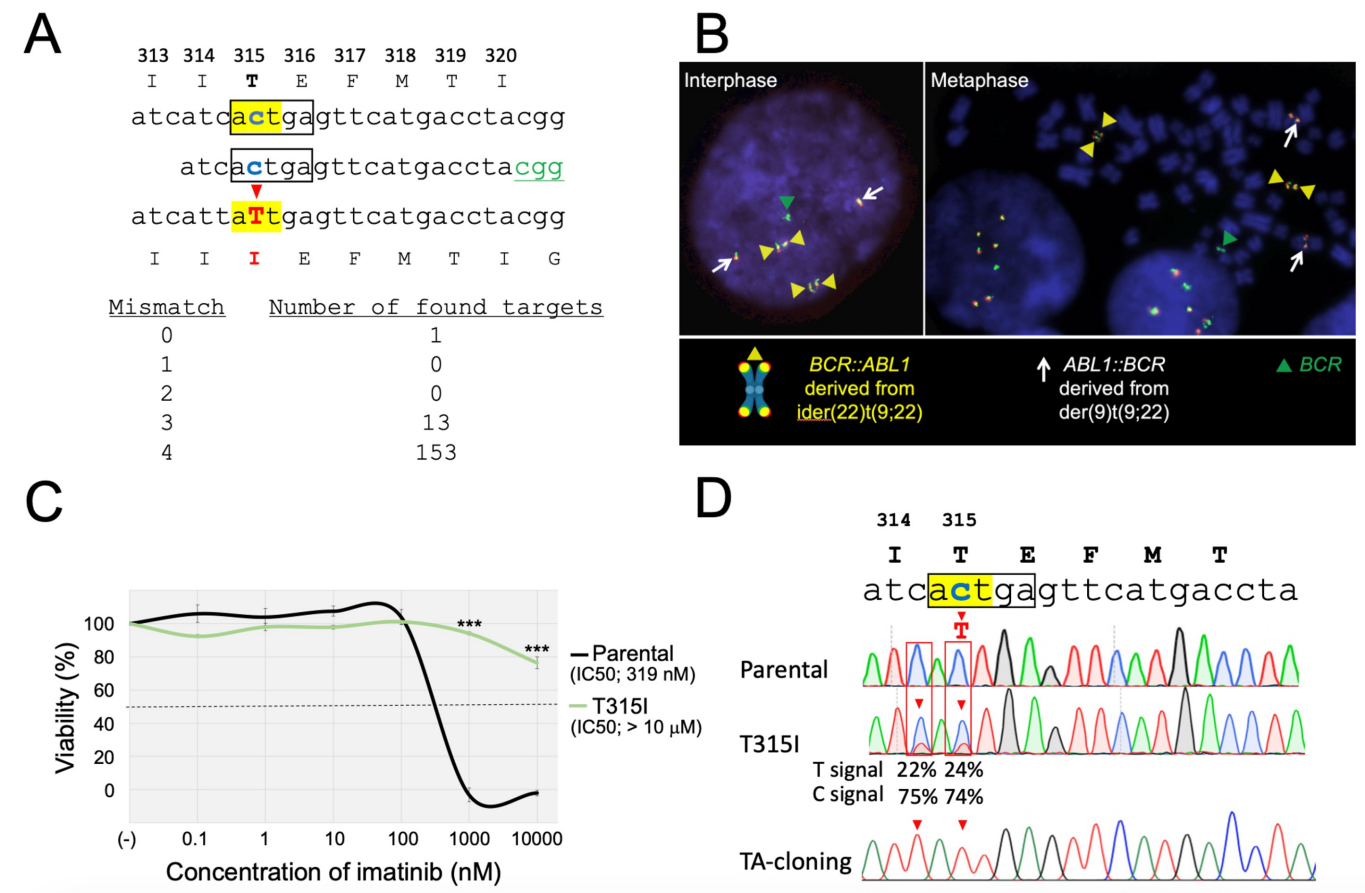
Introduction of T315I mutation into the BCR::ABL1 fusion gene of the TCCS cell line. A: Schematic representation of the sgRNA. Target codon 315 is highlighted in yellow. The PAM site is indicated in green and the editing window is indicated with a black box. The targeted C and converted T nucleotides are indicated in blue and red, respectively. Numbers of the potential off-target sites evaluated by Cas-OFFinder (http://www.rgenome.net/cas-offinder/) were indicated as follows. B: Interphase (left) and metaphase (right) fluorescence in situ hybridization analyses of parental cells using *ABL1-* and *BCR*-specific probes labeled with red and green fluorochromes, respectively. Yellow and white arrowheads indicate *BCR::ABL1* fusion signals derived from ider(22)t(9;22) and *ABL1::BCR* fusion signals derived from der(9)t(9;22), respectively, and green arrowheads indicate *BCR* signals. C: Dose-response curves of imatinib in the parental cells (black) and the T315I-subline (green). The horizontal and vertical axes indicate the imatinib concentration and cell viability, respectively. A representative dose-response curve of three independent assays, which were performed in triplicated analysis, was indicated. Error bars indicate the standard deviations of the triplicated analysis. An asterisk is indicated at each concentration when statistically significant (***p < 0.001 in the t-test). The IC_50_ values are indicated. D: Sanger sequencing of genomic PCR products in the parental cells (top), the T315I-subline (middle), and the mutated allele in the T315I-subline after TA-cloning (bottom). The intensities of T and C signals evaluated with EditR software are indicated.

First, to verify the utility of the CBE system (Figure 1), we tried to introduce the T315I mutation (ACT to ATT) of the *BCR::ABL1* fusion gene in a human Ph+ leukemia cell line. We designed a single guide RNA (sgRNA) with the protospacer adjacent motif (PAM) site (CGG) located 16 bp downstream of the targeted C nucleotide of codon 315 (Figure 2A). In this sgRNA, there were no potential off-target sites with one or two base mismatches. There are no other additional C nucleotides in the editing window (ACTGA) spanned from 13-17 nucleotides upstream of the PAM site [11]. We used the TCCS cell line, which has native *BCR::ABL1* fusion genes and is sensitive to TKIs including imatinib [10]. Dual-color fluorescence *in situ* hybridization analysis of the parental TCCS in the metaphase showed four fusion signals derived from the *BCR::ABL1 *fusion genes (Figure 2B). After electroporation of the CBE component, the cells were incubated in a 24-well plate. After one week of expansion, the cells were cultured with 1 µM of imatinib, which completely eradicated the parental cells within one week, and an imatinib-resistant subline was obtained after one week of selection. The IC_50_ value of imatinib in the obtained subline was an over 100-fold increase compared to that in parental cells (IC_50_ value; >10 μM vs. 319 nM) (Figure 2C). Direct Sanger sequencing of the genomic PCR product confirmed a small peak of the T nucleotide corresponding to the T315I mutation, in addition to the wild-type sequence (Figure 2D). Moreover, another small peak of T nucleotide corresponding to the silent mutation of codon 314 was observed, while no indels around the target site were observed. To confirm whether these two C-to-T transitions were simultaneously induced in the same allele, we also performed DNA sequencing analysis after TA cloning of the PCR amplicon from the subline. Sequencing analysis of each individual read confirmed that both C-to-T transitions were induced in the same allele. The signal intensities of C-to-T transitions in the DNA sequencing of the obtained imatinib-resistant subline were estimated as 22-24% in the EditR analysis. Since TCCS has four *BCR::ABL1* genes, these observations demonstrated that T315I and adjacent silent mutations were simultaneously introduced into one of four *BCR::ABL1* genes in the entire cell pool of the subline as a result of base edit.

TKI resistance in the T315I-subline

**Figure 3:**
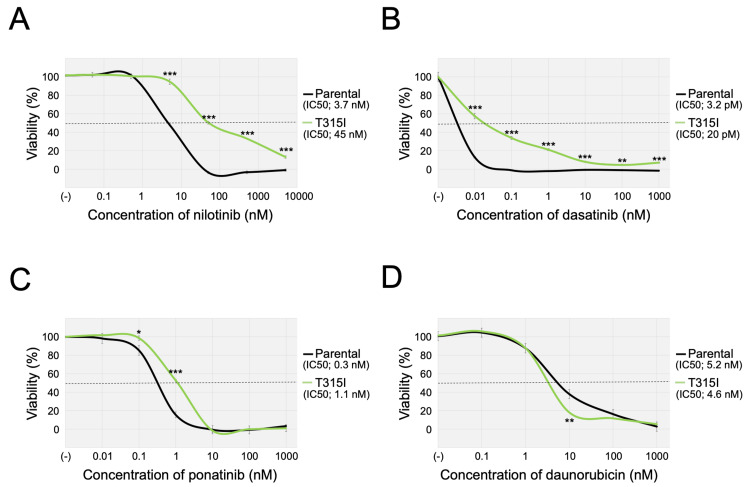
TKI sensitivities of the parental cells and the T315I-subline. A-D: Sensitivities of the parental cells (black lines) and the T315I-subline (green lines) to nilotinib (A), dasatinib (B), ponatinib (C), and daunorubicin (D). The horizontal and vertical axes indicate the drug concentration and cell viability, respectively. A representative dose-response curve of three independent assays, which were performed in triplicated analysis, was indicated. Error bars indicate the standard deviations of the triplicated analysis. An asterisk is indicated at each concentration when statistically significant (*p < 0.05, **p < 0.01, ***p < 0.001 in the t-test). The IC_50_ values are indicated. TKI, tyrosine kinase inhibitor

We next evaluated the TKI sensitivities of the T315I-subline using the alamarBlue assay. Compared with the parental cells, the T315I-subline was more resistant to the three other TKIs (Figure 3). The median IC_50 _values of each TKI in the parental cells and the T315I-subline in three independent analyses were as follows: nilotinib, 3.7 nM (range: 2.7 - 4.5 nM) vs. 45 nM (25 - 52 nM), p = 0.002 (Figure 3A); dasatinib, 3.2 pM (3.2 - 3.7 pM) vs. 59 pM (20 - 95 pM), p = 0.03 (Figure 3B); and ponatinib, 0.3 nM (0.1 - 0.5 nM) vs 1.1 nM (0.5-1.3 nM), p = 0.03 (Figure 3C). In contrast, the parental cells and the T315I-subline revealed similar sensitivities to daunorubicin: the median IC_50_ value was 4.6 nM (range: 3.3-7.6 nM) vs. 5.2 nM (4.3 - 5.7 nM), p = 0.32 (Figure 3D). These observations indicated that the monoallelic T315I mutation induced specific resistance to TKIs in the Ph-positive leukemia cell line with four intrinsic *BCR::ABL1* fusion genes.

Introduction of the T125M mutation of the *TP53* gene into the BCP-ALL cell line 697 

**Figure 4:**
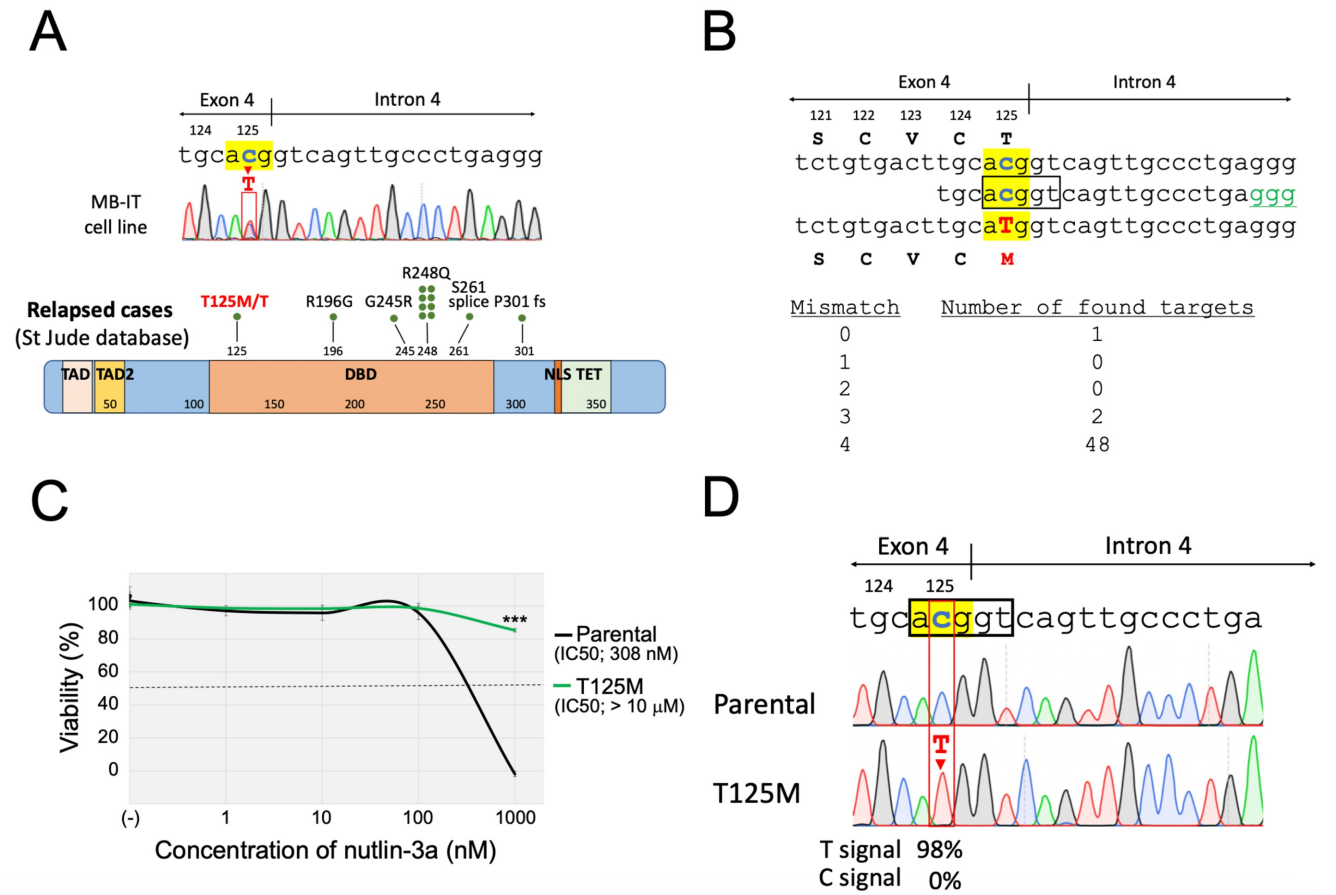
Introduction of the T125M mutation into the TP53 gene of the 697 cell line. A: T125M mutation of the *TP53* gene in MB-IT cell line and distribution of *TP53* mutations in relapsed ALL cases reported in the St. Jude database. In the top panel, direct Sanger sequencing of the genomic PCR product in the MB-IT cell line is indicated. Codon 125 is highlighted in yellow and the T125M mutation is indicated in the red box. B: Schematic representation of the sgRNA. Target codon 125 is highlighted in yellow. The window is indicated with a black box. The targeted C and converted T nucleotides are indicated in blue and red, respectively. Numbers of the potential off-target sites evaluated by Cas-OFFinder (http://www.rgenome.net/cas-offinder/) were indicated as follows. C: Dose-response curves of nutlin-3a in parental cells (black) and T125M-subline (green). The horizontal and vertical axes indicate the drug concentration and cell viability, respectively. A representative dose-response curve of three independent assays, which were performed in triplicated analysis, was indicated. Error bars indicate the standard deviations of the triplicated analysis. An asterisk is indicated at each concentration when statistically significant (***p < 0.001 in the t-test). The IC_50_ values are indicated. D: Sanger sequencing of the genomic PCR product in the parental cells (top) and the nutlin-3a-resistant subline (bottom). The intensities of T and C signals evaluated by the EditR software are indicated.

We then tried to introduce the specific mutation of the* TP53* gene by using the CBE system in a human BCP-ALL cell line. We recently identified the heterozygous T125M (ACG to ATG) mutation of the *TP53* gene (Figure 4A) in one of the BCP-ALL cell lines, MB-IT, which was established at diagnosis from a 10-year-old ALL patient [17]. Residue 125 is located in the DNA-binding domain and is reportedly involved in the formation of the hydrogen bond to the G117 and G282 residues [20]. Accordingly, the T125M mutation may disrupt the transcription activity of the p53 protein. To verify its significance in drug sensitivities, we utilized the CBE system for the introduction of the T125M mutation in a BCP-ALL cell line without the *TP53* mutation. We designed the sgRNA with the PAM site (GGG) located 16 bp downstream of the targeted C nucleotide of codon 125 (Figure 4B). In this sgRNA, there were no potential off-target sites with one or two base mismatches. There is no other C nucleotide in the corresponding editing window (ACGGT). We used the 697 cell line, which is relatively sensitive to most of the representative chemotherapeutic agents [15]. 697 has the intact *TP53* gene [16], and, subsequently, is highly susceptible to nutlin-3a, an inhibitor for MDM2 that plays an essential role in the ubiquitylation and proteasomal-dependent degradation of p53 protein [21]. After electroporation of the CBE component, the cells were incubated in a 24-well plate. After one week of expansion, the cells (IC_50_ value of nutlin-3a was approximately 0.3 µM) were incubated with nutlin-3a at 10 µM, which completely eradicated the parental cells within one week, and a nutlin-3a-resistant subline was obtained after two-week selection. The obtained subline was resistant to nutlin-3a (IC_50_ value; > 1 μM) (Figure 4C). Direct Sanger sequencing performed in the genomic PCR product confirmed the acquisition of the homozygous T125M mutation with an editing efficiency of 98% (Figure 4D). No other C-to-T transitions or indels were observed in the adjacent areas. These observations suggested that the nutlin-3a-resistant subline acquired the homozygous T125M mutation of the intrinsic *TP53* gene as a result of base edit without acquiring indels.

Disruption of p53 transcriptional activities in the T125M-subline

**Figure 5:**
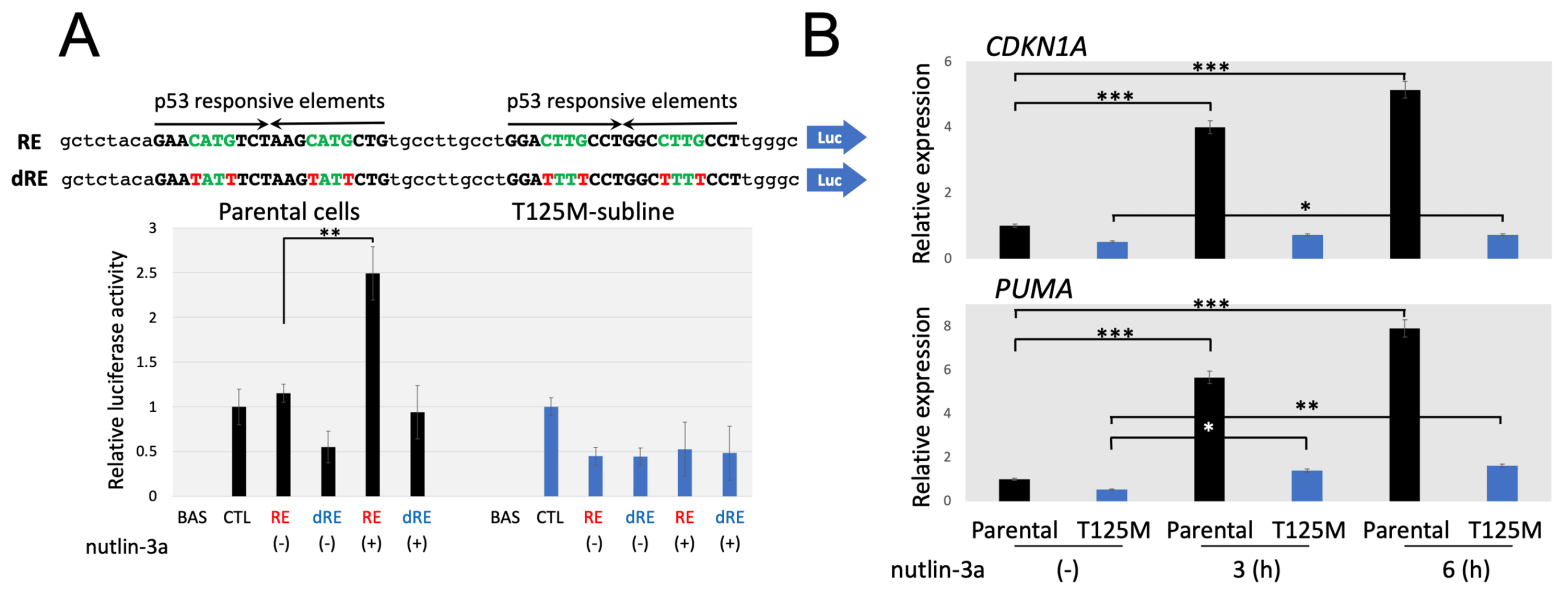
Transactivation activity of the p53 protein in the parental cells and the T125M-subline. A: Reporter assay of the p53 protein. Sequences of wild-type and mutated p53 responsive elements in the pGL3-RE (upper) and pGL3-dRE (lower) plasmids are indicated, respectively, at the top. Relative luciferase activities in the parental cells (left) and the T125M-subline (right) cultured in the presence (+) or absence (-) of nutlin-3a are indicated at the bottom. The luminescence produced by *Renilla luciferase* was used as an internal standard. BAS, pGL3-basic; CTL, pGL3-control. Asterisk is indicated when statistically significant (**p < 0.01 in the t-test). B: Changes in the gene expression levels of the *CDKN1A *(upper panel) and *PUMA* (lower panel). Real-time RT-PCR analysis was performed in the parental cells (black column) and the T125M-subline (blue column) cultured in the presence or absence of nutlin-3a for the indicated periods using the *ACTB* gene expression level as an internal control. Error bars indicate the standard deviation in triplicated analyses. Asterisks are indicated when statistically significant (*p < 0.05, **p < 0.01, ***p < 0.001 in the t-test).

We first evaluated the significance of the T125M mutation on the transcriptional activity of the p53 protein using luciferase assay (Figure 5A). We transfected either the reporter plasmid carrying the p53-responsive elements (pGL3-RE) or the disrupted p53-responsive elements (pGL3-dRE), and the cells were incubated for 24 h. We also transfected pGL3-basic and pGL3-control as negative and positive control. Then, the cells were treated or untreated with 10 µM of nutlin-3a for 6 h and harvested for the luciferase assay. Almost similar activities were observed in the parental cells transfected with pGL3-RE and in those transfected with the pGL3-control. Moreover, after the nutlin-3a treatment, significantly upregulated activities were observed in the parental cells transfected with pGL3-RE but not in those transfected with pGL3-dRE. In contrast, in the T125M-subline, luciferase activities remained relatively low in the pGL3-RE and pGL3-dRE-transfected subline compared with the pGL3-control-transfected subline, even after the nutlin-3a treatment. We next evaluated the gene expression levels of representative downstream targets of the p53 protein. The cells were treated with or without nutlin-3a at 5 µM for 6 h, and real-time RT-PCR analyses were performed (Figure 5B). In the parental cells, the gene expression levels of both *CDKN1A *(involved in p53-mediated cell cycle arrest) and *PUMA* (involved in p53-mediated apoptosis) were markedly upregulated by nutlin-3a-treatment. Meanwhile, *CDKN1A* and *PUMA* levels were almost unchanged in the T125M-subline by the nutlin-3a treatment. These observations indicated that the T125M mutation of the *TP53* gene significantly disrupted p53 transactivation activity.

Reduced sensitivity to chemotherapeutic drugs and irradiation in the T125M-subline

**Figure 6:**
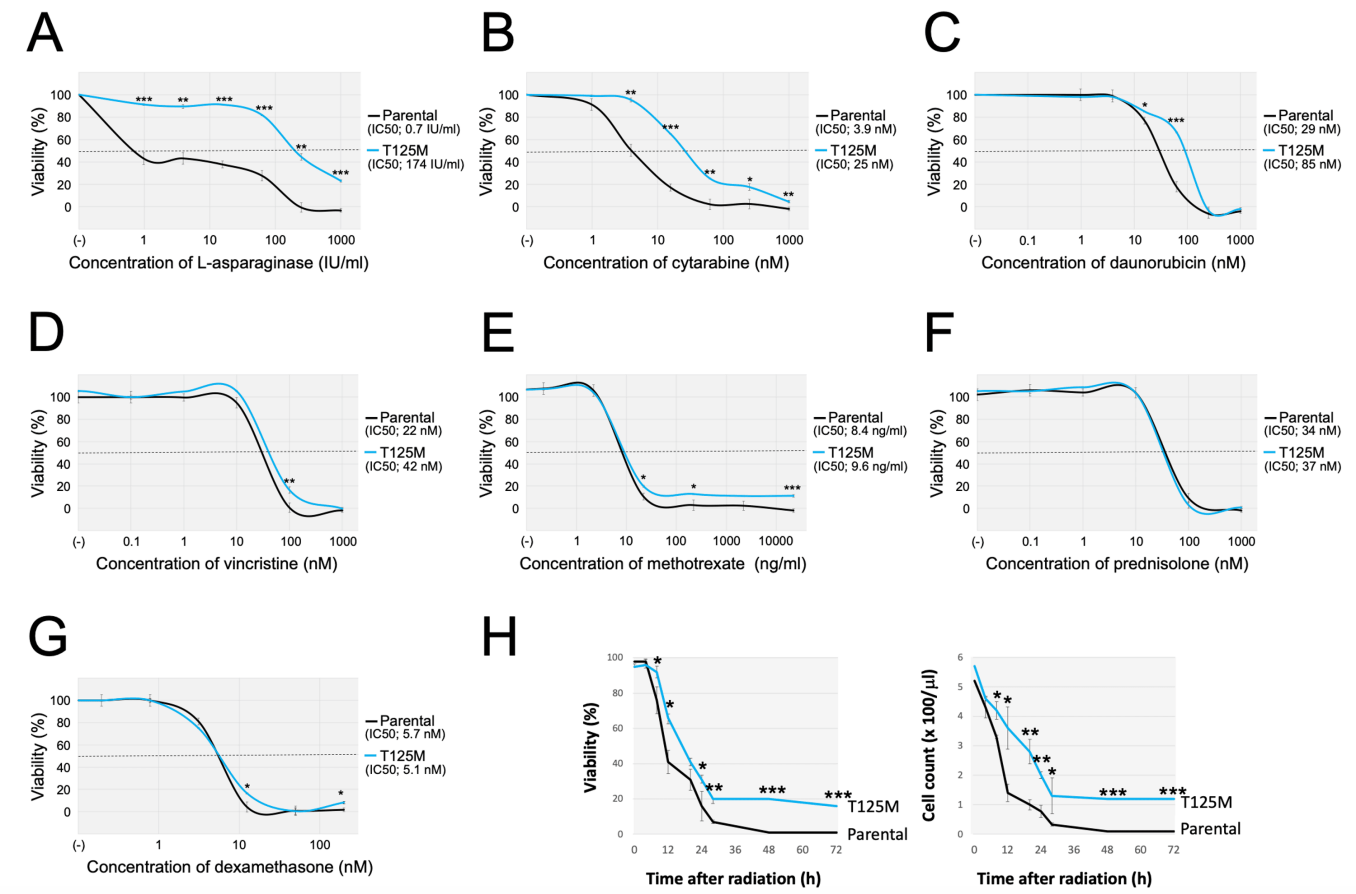
Sensitivities to representative chemotherapeutic agents and irradiation of the parental cells and the T125M-subline. A-G: Dose-response curves of asparaginase (A), cytarabine (B), daunorubicin (C), vincristine (D), methotrexate (E), prednisolone(F), and dexamethasone (G) in the parental cells (black lines) and the T125M-subline (blue lines). The horizontal and vertical axes indicate the drug concentration and cell viability, respectively. Representative dose-response curve of three independent assays, which were performed in triplicated analysis, was indicated. Error bars indicate the standard deviations of the triplicated analysis. An asterisk is indicated at each concentration when statistically significant (*p < 0.05, **p < 0.01, ***p < 0.001 in the t-test). The IC_50_ values are indicated. H: Time course of cell viability (left panel) and viable cell count (right panel) after 10 Gy radiation in the parental cells (black lines) and the T125M-subline (blue lines). Error bars indicate the standard deviations of triplicated analysis. An asterisk is indicated at the time point when statistically significant (*p < 0.05, **p < 0.01, ***p < 0.001 in the t-test).

We evaluated the significance of the T125M mutation in the drug sensitivities to representative chemotherapeutic agents and irradiation. We first evaluated the drug sensitivities to seven representative agents using an alamarBlue assay. Of note, the T125M-subline was significantly more resistant to asparaginase (Figure 6A) and cytarabine (Figure 6B) compared with the parental cells. Moreover, the T125M-subline tended to be more resistant to daunorubicin (Figure 6C). In contrast, the T125M-subline was almost equally sensitive to vincristine (Figure 6D), methotrexate (Figure 6E), prednisolone (Figure 6F), and dexamethasone (Figure 6G) as the parental cells. Finally, we investigated radiation sensitivity. After 10 Gy irradiation, the cellular viability was determined by the trypan blue exclusion assay. In both cell viabilities and living cell numbers, the T125M-subline was significantly more resistant to irradiation than the parental cells (Figure 6H). These observations indicated that the introduction of the T125M mutation into the *TP53* gene in a BCP-ALL cell line reduced sensitivities to several agents and irradiation.

## Discussion

In this study, we applied the CBE system and successfully introduced the T315I mutation into one of four *BCR::ABL1* alleles in the TCCS cell line. Application of the CBE system for introducing the T315I mutation of the *BCR::ABL1* gene is reasonable since cytidine deaminase itself was reportedly an intrinsic mechanism for the induction of the T315I mutation in CML [22]. In the CBE system, the canonical editing window is reported to be located in 13-17 nucleotides upstream of the PAM site [11]. Thus, the targeted C nucleotide located at codon 315 (ACT) was the only candidate for the C-to-T transition. Nevertheless, a silent C-to-T transition was additionally introduced at adjacent codon 314 (ATC) in the same allele, suggesting that the targeting scope of the C-to-T transition might be slightly expanded.

The obtained T315I-subline was more resistant to all tested TKIs (but not to daunorubicin) compared with the parent cells, although the T315I mutation happened to be introduced into one of four *BCR::ABL1* alleles. In this context, the BCR::ABL1 protein reportedly acts as a homotetramer [23,24]. Accordingly, in our T315I-subline, mutated BCR::ABL1 proteins may form tetramers with unmutated BCR::ABL1 proteins. Our observations suggest that T315I-acquired BCR::ABL1 proteins may disrupt the activity of BCR::ABL1 tetramers in a dominant-negative fashion, although further biochemical or structural validations are required. 

We then utilized the CBE system for the introduction of the T125M mutation of the *TP53* gene in the 697 cell line and successfully obtained a nutlin-3a-resistant subline, in which the T125M mutation was homozygously introduced. Of note, no additional C-to-T transitions were observed even though another C nucleotide is located at the adjacent codon 124 (TGC), suggesting that the width of the editing window in the CBE system might be slightly different in a cell-type-specific and/or target-specific manner. Moreover, the imatinib-resistant subline of TCCS cells acquired T315I mutation in only one of the four *BCR::ABL1* alleles, while the nutrin3a-resistant subline of 697 cells acquired T125M mutation in both of the two *TP53* alleles, indicating target-dependent variability in editing efficiencies of the CBE system in human leukemia cell lines. However, when any specific selection markers are available (such as imatinib for T315I mutation of *BCR::ABL1* and nutrin-3a for *TP53* mutations), the CBE system is useful for establishing human leukemia models with specific gene mutations even if its editing efficiencies are relatively low.

Clinically, the TP53 mutation is associated with drug resistance and poor prognosis in relapsed childhood ALL cases [25]. Several mutations at codon 125, including the T125R mutation [26] and the T125T splice-altering mutation [1], have been previously identified in ALL clinical samples. Codon 125 is encoded in the 5’ end of the DNA-binding domain, and we confirmed a significant disruption in the p53-mediated transactivation activity using the luciferase assay and the RT-PCR analysis of target gene expression. Of note, the T125M-subline was significantly more resistant to asparaginase and cytarabine, as well as irradiation. Thus, our T125M-subline will be a useful cellular model for developing new therapeutic modalities to overcome poor prognosis in *TP53*-mutated BCP-ALL cases.

Our observations demonstrated the utility and accuracy of the CBE system in introducing the specific C-to-T (G-to-A) conversion in human leukemic cell lines, although precise evaluation for potential off-target edits using whole genome sequencing had not been directly performed. The CBE system has a great advantage as a DSB-free genome editor by using a nCas9 [11]. Moreover, the CBE system induces nucleotide conversion by exogenously introducing cytidine deaminase activities without the intrinsic cellular DNA repair system, which could be largely disrupted in most leukemia cell lines [16]. However, editing efficiency for introducing the T315I mutation into TCCS was relatively low since only one of four *BCR::ABL1* genes acquired the mutation. In contrast, in our previous attempt to introduce the T315I mutation into TCCS via HDR [10], the obtained imatinib-resistant subline acquired the T315I mutation in all of the four *BCR::ABL1* genes.

Despite the above utilities, the base editing system has several major limitations. First, although the adenosine base editor and other base editors enable the generation of specific A-to-G and C-to-G conversions [11,27], the base editing system is unable to apply for the introduction of the other types of mutation. Second, the introduction of nucleotide conversion by the base editing system is limited within the editing window, although modification of deaminase activities could alter the width of the editing window [28]. To overcome these limitations in nucleotide conversion by the base editing system, the prime editing (PE) system has been newly established [29]. The PE system utilizes a reverse-transcriptase-fused nCas9 to directly introduce new sequences, which are embedded in the prime editing guide RNA (pegRNA) as a reverse transcriptase template, without inducing DSB [29]. 

Recently, by using the PE system, we tried to introduce the R248Q mutation (CGG>CAG) of the *TP53* gene, which is reported to be the most frequent relapse-specific *TP53* gene mutation in BCP-ALL cases [30], into the 697 cell line, since it is not inducible by the existing BE systems. Unexpectedly, although the targeted R248Q mutation was transduced as a result of prime edit, diverse types of unintended insertions derived from the scaffold sequence of the pegRNA were frequently integrated [31]. These observations suggest that the PE system might not be reliable for introducing at least several specific gene mutations in leukemic cell lines. 

## Conclusions

In the present study, we successfully introduced the T315I (ACT to ATT) mutation of the *BCR::ABL1* fusion gene and the T125M (ACG to ATG) mutation of the *TP53* gene into human leukemia cell lines by utilizing the CBE system and evaluated their significance in the drug sensitivities. Our observations demonstrated the utility of the CBE system for introducing specific C-to-T transition into the intrinsic gene of human leukemia cell lines, which could accelerate the development of novel targeted therapies for high-risk patients by establishing drug-resistant variant models. Moreover, base-edited leukemia models might be also useful to uncover a linkage between genetic mutations and the pathophysiology of leukemia. Ongoing functional modifications of the BE system to strengthen the diversity of the editing window could expand the spectrum of applicable gene mutations for studying drug resistance in leukemia and (presumably) diverse types of cancer.
